# Increased production of functional small extracellular vesicles in senescent endothelial cells

**DOI:** 10.1111/jcmm.15047

**Published:** 2020-02-26

**Authors:** Jaime A. Riquelme, Kaloyan Takov, Concepción Santiago‐Fernández, Xavier Rossello, Sergio Lavandero, Derek M. Yellon, Sean M. Davidson

**Affiliations:** ^1^ The Hatter Cardiovascular Institute University College London London UK; ^2^ Advanced Center for Chronic Diseases (ACCDiS) Facultad de Ciencias Quimicas y Farmaceuticas & Facultad de Medicina Universidad de Chile Santiago Chile; ^3^ Department of Gastroenterology Virgen de la Victoria University Hospital Instituto de Investigación Biomédica de Málaga (IBIMA) University of Malaga Malaga Spain; ^4^ Cardiology Division Department of Internal medicine University of Texas Southwestern Medical Center Dallas TX USA

**Keywords:** endothelium, exosomes, extracellular vesicles, senescence

## Abstract

Small extracellular vesicles (EVs) are novel players in vascular biology. However, a thorough understanding of their production and function remains elusive. Endothelial senescence is a key feature of vascular ageing and thus, is an attractive therapeutic target for the treatment of vascular disease. In this study, we sought to characterize the EV production of senescent endothelial cells. To achieve this, Human Umbilical Vascular Endothelial Cells (HUVECs) were replicated until they reached senescence, as determined by measurement of Senescence‐Associated β‐Galactosidase activity via microscopy and flow cytometry. Expression of the endosomal marker Rab7 and the EV marker CD63 was determined by immunofluorescence. Small EVs were isolated by ultracentrifugation and characterized using electron microscopy, nanoparticle tracking analysis and immunoassays to assess morphology, size, concentration and expression of exosome markers CD9 and CD81. Migration of HUVECs in response to EVs was studied using a transwell assay. The results showed that senescent endothelial cells express higher levels of Rab7 and CD63. Moreover, senescent endothelial cells produced higher levels of CD9‐ and CD81‐positive EVs. Additionally, small EVs from both young and senescent endothelial cells promoted HUVEC migration. Overall, senescent endothelial cells produce an increased number of functional small EVs, which may have a role in vascular physiology and disease.

## 1 INTRODUCTION

Small extracellular vesicles (sEVs) are novel players in vascular biology. The possibility that sEVs may act as an intercellular communication mechanism has attracted substantial attention in cardiovascular research, given their potential role as either biomarkers or therapeutic agents.^1^ Various studies have suggested an important functional role for endothelial‐derived sEVs in the context of cardiovascular physiology and pathophysiology.^2^, ^3^ Moreover, there is an increasing amount of evidence showing that the production and function of sEVs may vary in response to protective/deleterious stimuli or different physiological states.^1^, ^4^, ^5^ In this context, endothelial senescence is a key feature of vascular ageing and thus, it is an attractive therapeutic target for the treatment of vascular disease. Considering that during ageing, the number of sEVs may be altered and their function impaired,^6^ we sought to characterize the production and function of sEVs in the setting of endothelial senescence, in order to provide insights that may eventually contribute to the understanding of their role in cardiovascular physiology and disease.

## 2 METHODOLOGY

### 2.1 Cell culture and senescence assessment

Human umbilical vein endothelial cells (HUVECs) from Lonza were cultured in Endothelial Basal Medium‐2 supplemented with EGM‐2 SingleQuot Kit and used as passage 3‐12 for young cells or 18‐22 for senescent cells. Senescence was measured by senescence‐associated β‐galactosidase (SA β‐galactosidase) Staining Kit (Cell Signalling Technology) and by flow cytometry using C12FDG (5‐dodecanoylaminofluorescein Di‐β‐D‐galactopyranoside (Thermo Fisher Scientific; 33 μmol/L) and chloroquine pretreatment (300 μmol/L),^7^ using an Accuri C6 flow cytometer (BD Biosciences).

### 2.2 Western blot analysis

Proteins from HUVEC cell lysates were separated by electrophoresis on 4%‐20% gradient gels (Thermo Fisher Scientific) and transferred onto nitrocellulose membranes (GE Healthcare Life Sciences) via wet transfer. Membranes were probed for p16 (10883‐1‐AP, Proteintech) using GAPDH (9484 Abcam) as loading control. Intensity of fluorescently conjugated secondary antibodies was quantified using Odyssey Imaging System (LiCor Biosciences).

### 2.3 Assessment of lysosomes

For electron microscopy, cells were fixed with 1.5% gluteraldyhyde/2% paraformaldehyde in 0.1 mol/L phosphate buffer (pH 7.3), osmicated in 1% OsO_4_/0.1 mol/L phosphate buffer, dehydrated in a graded ethanol‐water series, cleared in propylene oxide and infiltrated with Araldite resin. Ultrathin sections were cut onto 300 mesh grids, stained with lead citrate and imaged on a Jeol 1010 transmission electron microscope. For confocal microscopy, young and senescent HUVECs were stained with LysoSensor™ Green DND‐189 (Thermo Fisher Scientific) and imaged on a Leica TCS SP5 confocal microscope.

### 2.4 Immunofluorescence

Young and senescent HUVECs were cultured on a µ‐Slide (ibidi), fixed with 4% paraformaldehyde and permeabilized with 0.1% Triton X‐100. Immunofluorescence staining was performed for RAB7 (9367, Cell signalling Technology) and CD63 (556019, BD Bioscience), with Hoechst 33258 (10 µg/mL) for nuclear labelling. Images were taken using a Leica TCS SP5 confocal microscope.

### 2.5 Small extracellular vesicle isolation

Three T75 flasks of young or senescent HUVECs were incubated for 48 hours in medium containing exosome‐free FBS (Thermo Fisher Scientific). Conditioned medium was then collected and centrifuged at 300 *g* (10 minutes, 4°C), 2000 *g* (10 minutes, 4°C) and 10 000 *g* (30 minutes, 4°C). The supernatants were then ultracentrifuged at 100 000 *g* (70 minutes, 4°C) using Optima MAX‐XP ultracentrifuge (Beckman Coulter) and MLA‐55 rotor, including a PBS wash step.^8^

### 2.6 Small extracellular vesicle analysis

Nanoparticle tracking analysis (NTA) was performed on a NanoSight LM10‐HS (Malvern) using 488‐nm laser module and NTA 3.1 software.^9^ Constant flow injection was used, and 3‐5 videos of 30 seconds were captured with Camera Level of 15 and Detection Threshold of 4. Exosome‐specific markers (CD9: antibody 555370 and CD81: antibody 555675, BD Biosciences) were quantified using a previously described Dissociation‐enhanced lanthanide fluorescence immunoassay (DELFIA).^10^, ^11^, ^12^ sEVs were visualized by electron microscopy after a standard staining procedure with 0.5% uranyl acetate.^8^

### 2.7 Endothelial cell migration

A modified Boyden's chamber assay^13^ was performed to assess for pro‐migratory functions of sEV isolates on HUVECs. sEVs or vehicle (PBS) were added to the bottom wells of a 12‐well chemotaxis chamber (NeuroProbe) at the indicated concentrations. About 30,000 HUVECs/well in Endothelial Serum‐Free Defined Medium (Cell Applications Inc) were added to the top wells of the chamber, and an 8‐µm pore polycarbonate track‐etch membrane (NeuroProbe) was used as a barrier. Chambers were incubated for 6 hours, and membranes were collected, cells were fixed with cold methanol, stained using 0.5% (w/v) crystal violet solution and scanned on CanoScan LiDE 220 scanner (Cannon). Staining intensity of each well was quantified using ImageJ.

### 2.8 Statistical analysis

Bar graphs show mean ± SEM. Data were compared by Student's *t* tests or one‐way ANOVA with Tukey's post hoc test using GraphPad Prism 5 (GraphPad). A value of *P* < .05 was considered statistically significant.

## 3 RESULTS

### 3.1 Characterization of endothelial cell senescence

**Figure 1:**
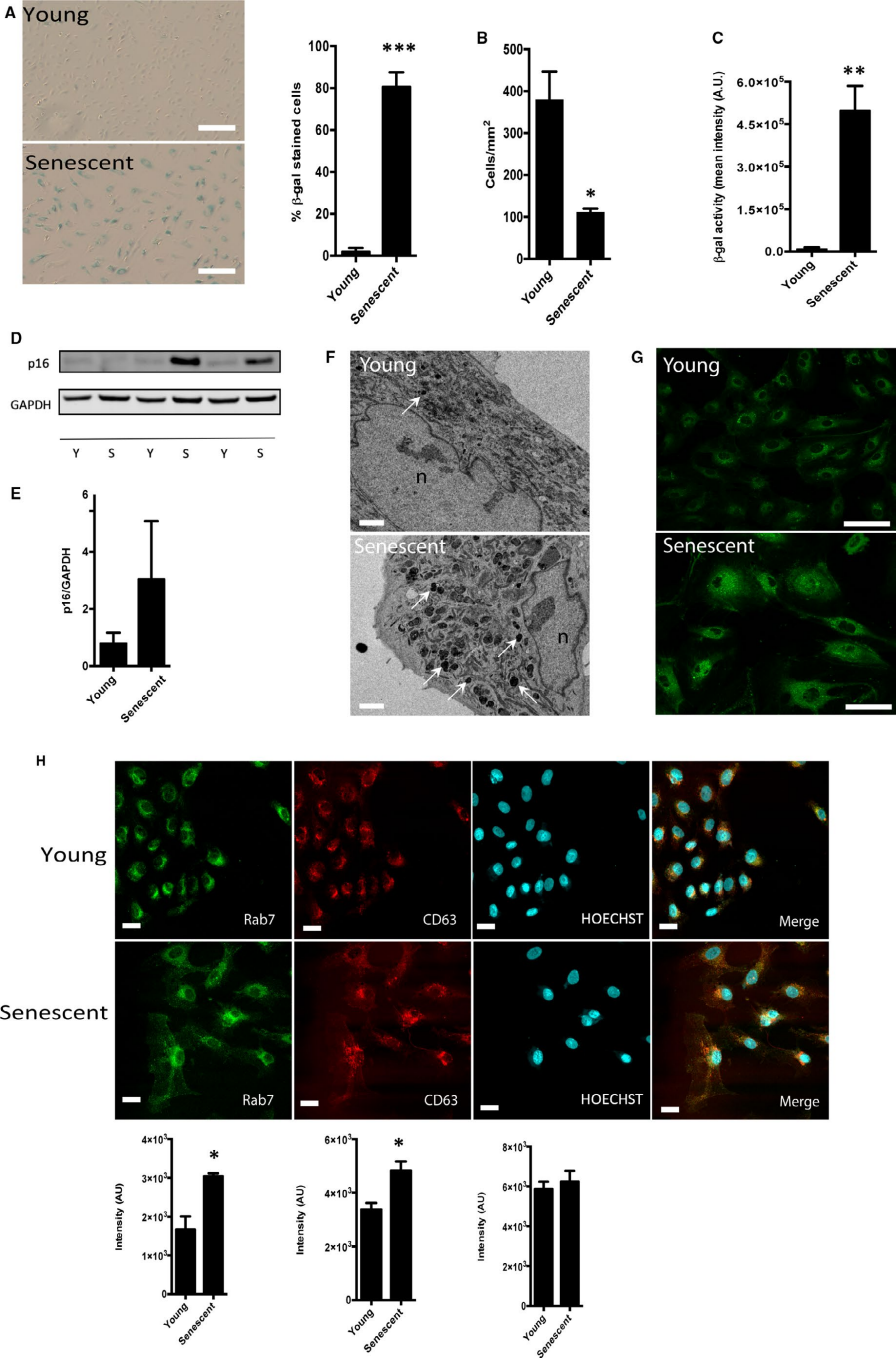
Characterization of senescence in HUVECs. (A) HUVECs were grown to passage 3‐12 and 18‐22 to compare the ‘young’ vs ‘senescent’ phenotype, respectively. β‐galactosidase staining was assessed by optical microscopy (N = 3). Scale bar 200 μm. (B) Cell number of young and senescent HUVECs was quantified per mm^2^ (N = 3). (C) β‐galactosidase activity was measured by flow cytometry (N = 3). (D, E) Representative image and densitometry of p16 expression by Western blot in young and senescent endothelial cells (N = 7). Representative images showing increased lysosomes in senescent cells assessed by (F) electron microscopy and (G) LysoSensor (1 μmol/L) staining using confocal microscopy. White arrows show electron‐dense spots compatible with lysosome morphology (n: nucleus). Scale bar 5 and 75 μm for electron and confocal microscopy, respectively. (H) Representative images showing immunofluorescence of young and senescent HUVECs stained with RAB7, CD63 and Hoechst in upper panel, with the correspondent quantification of fluorescence in the lower panel (N = 3). Scale bar 25 μm. *P ˂ .05 vs young, **P ˂ .001 vs young, ***P ˂ .0001 vs young. Bar graphs represent mean ± SEM. Data were analysed by unpaired, two‐tailed Student's *t* test

Primary HUVECs were cultured continuously until the cells ceased replication (passages 18‐22). Senescence was identified by assessing SA β‐galactosidase staining in these cells compared with control HUVECs, which had been cultured for 3‐12 passages (Figure 1A). Successful establishment of the senescent phenotype was further verified by the typical large, flattened morphology of the HUVEC, with significantly fewer cells present in a confluent dish of senescent HUVECs in comparison with young HUVECs (Figure 1A,B). Increased SA β‐galactosidase activity was confirmed using flow cytometry (Figure 1C). We then evaluated the p16 protein levels, which showed a strong but inconsistent increase in some late passage cells by Western blotting (Figure 1D,E). Moreover, lysosomes observed with electron and confocal microscopy were increased in number in senescent cells in comparison with young ones (Figure 1F,G). Hereon, we refer to the cells from early and late passages as ‘young’ and ‘senescent’, respectively.

### 3.2 The production of functional sEVs is increased in senescent endothelial cells

**Figure 2:**
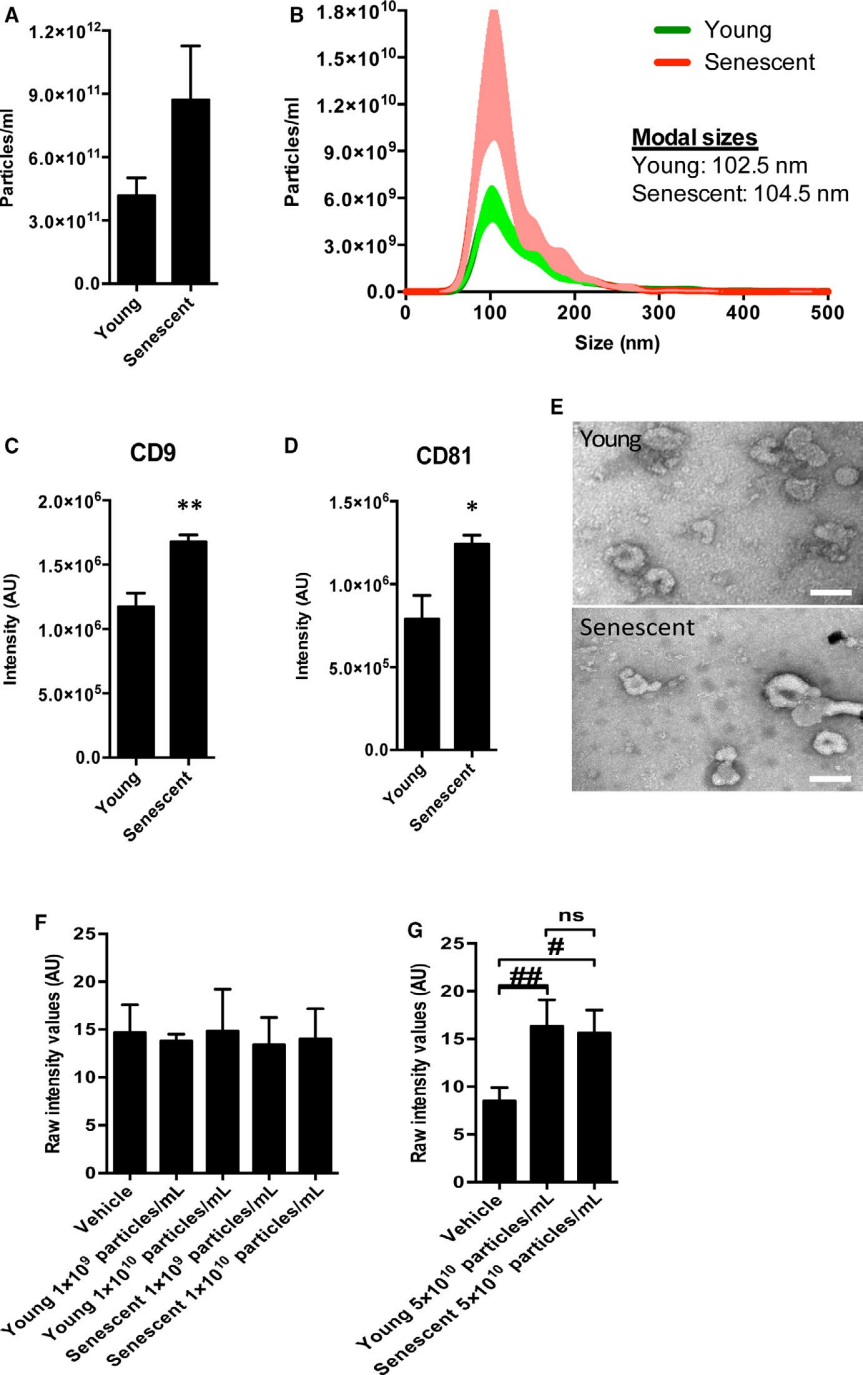
Characterization and assessment of function of extracellular vesicles produced by senescent HUVECs. Particle concentration (A) and size (B) were measured by nanoparticle tracking analysis in extracellular vesicles isolated from young and senescent HUVECs (N = 4‐8). Expression of CD9 (C) and CD81 (D) in sEVs of young and senescent HUVECs was determined by DELFIA inmmunoassay (N = 4‐8). (E) Representative images of sEVs isolated from young and senescent HUVECs. Scale bar 100 nm. Young HUVECs were treated with low (F) and high (G) concentrations of sEVs isolated from young and senescent HUVECs, and migration was assessed using a modified Boyden's chamber assay (N = 3 for F and N = 6 for G). *P ˂ .05 vs young, **P ˂ .05 vs young, ^#^ *P* ˂ .05 vs vehicle, ^##^ *P* ˂ .001 vs vehicle. Bar graphs represent mean ± SEM. Data were analysed by unpaired, two‐tailed Student's *t* test (A, C, D) and one‐way ANOVA, followed by Tukey's post test for multiple comparisons (F and G)

To evaluate whether senescence affects expression of the protein machinery for the production of sEVs, HUVECs were immunostained for the late endosomal marker RAB7 and the exosomal protein CD63. Senescent cells were found to have higher intracellular expression of both RAB7 and CD63 in comparison with young cells (Figure 1H). sEVs were then isolated from HUVEC‐conditioned medium by differential ultracentrifugation. Nanoparticle tracking analysis showed no significant differences in size and concentration of sEVs derived from young or senescent HUVECs (Figure 2A,B). However, an immunoassay specific for the exosome marker proteins CD9 and CD81 revealed significantly greater expression in senescent sEVs (Figure 2C,D). Transmission electron microscopy demonstrated that both young and senescent sEVs have the typical morphology of exosomes (Figure 2E).^8^ Finally, we evaluated whether sEVs from senescent endothelial cells were functional. We have previously shown that sEVs can act as a chemoattractant in a cell migration assay.^12^ Here, HUVECs were exposed to young or senescent sEVs at both low and high concentrations. sEVs from both young and senescent cells induced migration at high, but not at low concentrations of vesicles (Figure 2F,G).

## 4 DISCUSSION

The potential role of sEVs in vascular health and disease is suggested by the interesting proposal that their production and content may represent a ‘snapshot’ of the physiological state of a cell or tissue.^1^ In this context, cellular senescence is a naturally occurring process related to ageing and as such, elicits multiple changes in cell function.^14^ Senescent cells can potentially affect their microenvironment by producing altered autocrine and paracrine signals, which may be attributed to their senescence‐associated secretory phenotype (SASP).

Our study showed that while senescent endothelial cells did not generate a higher number of particles/mL as measured by NTA, they had an increased expression of the multivesicular body protein RAB7 and the tetraspanin CD63, indicative of an increased facility for the production of sEVs. This observation was confirmed by the increase in exosome markers CD9 and CD81 measured in the isolated senescent sEVs. We have previously reported that certain conditions, such as in vitro simulated ischaemic preconditioning, may affect rates of sEV production.^3^ Thus, future investigations should seek to study the functional consequences of the physiological and pathophysiological alterations of the endothelial production of sEVs, as well as to accurately determine their content.

Our work also reveals that at low concentrations, sEVs isolated from both young and senescent endothelial cells are unable to induce migration of endothelial cells. At higher concentrations, both types of sEVs promoted migration to the same degree. This finding indicates that senescence does not appear to impair the functional capabilities of the secreted sEVs. However, given that senescent cells produce more sEVs, it is possible this equates to a greater pro‐migratory potential. It will be important to evaluate this potential in vivo in the future.

Taken together, our results suggest that senescent endothelial cells produce more, functional sEVs, which may impact vascular physiology and disease.

## CONFLICT OF INTEREST

The authors declare no conflict of interest.

## AUTHOR CONTRIBUTIONS

All authors made substantial contributions to research design, or the acquisition, analysis or interpretation of data; drafting the paper or revising it critically; and approval of the submitted and final versions.

## Data Availability

The data that support the findings of this study are available from the corresponding author upon reasonable request.

## References

[1] Yellon DM , Davidson SM . Exosomes: nanoparticles involved in cardioprotection? Circ Res. 2014;114:325‐332. 24436428 10.1161/CIRCRESAHA.113.300636

[2] Brahmer A , Neuberger E , Esch‐Heisser L , et al. Platelets, endothelial cells and leukocytes contribute to the exercise‐triggered release of extracellular vesicles into the circulation. J Extracell Vesicles. 2019;8:1615820. 31191831 10.1080/20013078.2019.1615820 PMC6542154

[3] Davidson SM , Riquelme JA , Zheng Y , et al. Endothelial cells release cardioprotective exosomes that may contribute to ischaemic preconditioning. Sci Rep. 2018;8:15885. 30367147 10.1038/s41598-018-34357-z PMC6203728

[4] de Jong OG , Verhaar MC , Chen Y , et al. Cellular stress conditions are reflected in the protein and RNA content of endothelial cell‐derived exosomes. J Extracell Vesicles. 2012;1:18396. 10.3402/jev.v1i0.18396 PMC3760650 24009886

[5] Gollmann‐Tepekoylu C , Polzl L , Graber M , et al. miR‐19a‐3p containing exosomes improve function of ischemic myocardium upon shock wave therapy. Cardiovasc Res. 2019 pii: cvz209. 10.1093/cvr/cvz209. [Epub ahead of print] 31410448

[6] Kadota T , Fujita Y , Yoshioka Y , et al. Emerging role of extracellular vesicles as a senescence‐associated secretory phenotype: insights into the pathophysiology of lung diseases. Mol Aspects Med. 2018;60:92‐103. 29146100 10.1016/j.mam.2017.11.005

[7] Abbas M , Jesel L , Auger C , et al. Endothelial microparticles from acute coronary syndrome patients induce premature coronary artery endothelial cell aging and thrombogenicity: role of the Ang II/AT1 receptor/NADPH oxidase‐mediated activation of mapks and pi3‐kinase pathways. Circulation. 2017;135:280‐296. 27821539 10.1161/CIRCULATIONAHA.116.017513

[8] Thery C , Amigorena S , Raposo G , et al. Isolation and characterization of exosomes from cell culture supernatants and biological fluids. Curr Protoc Cell Biol. 2006;Chapter 3:Unit 3 22. 10.1002/0471143030.cb0322s30 18228490

[9] Gardiner C , Ferreira YJ , Dragovic RA , et al. Extracellular vesicle sizing and enumeration by nanoparticle tracking analysis. J Extracell Vesicles. 2013;2:19671. 10.3402/jev.v2i0.19671 PMC3760643 24009893

[10] Welton JL , Webber JP , Botos LA , et al. Ready‐made chromatography columns for extracellular vesicle isolation from plasma. J Extracell Vesicles. 2015;4:27269. 25819214 10.3402/jev.v4.27269 PMC4376847

[11] Takov K , Yellon DM , Davidson SM . Confounding factors in vesicle uptake studies using fluorescent lipophilic membrane dyes. J Extracell Vesicles. 2017;6:1388731. 29184625 10.1080/20013078.2017.1388731 PMC5699187

[12] Takov K , Yellon DM , Davidson SM . Comparison of small extracellular vesicles isolated from plasma by ultracentrifugation or size‐exclusion chromatography: yield, purity and functional potential. J Extracell Vesicles. 2019;8:1560809. 30651940 10.1080/20013078.2018.1560809 PMC6327926

[13] Boyden S . The chemotactic effect of mixtures of antibody and antigen on polymorphonuclear leucocytes. J Exp Med. 1962;115:453‐466. 13872176 10.1084/jem.115.3.453 PMC2137509

[14] Lopez‐Otin C , Blasco MA , Partridge L , et al. The hallmarks of aging. Cell. 2013;153:1194‐1217. 23746838 10.1016/j.cell.2013.05.039 PMC3836174

